# Miescher’s Cheilitis as a Diagnostic and Therapeutic Challenge—A Case Report

**DOI:** 10.3390/medicina61020299

**Published:** 2025-02-09

**Authors:** Katarzyna Błochowiak, Aya Kraiz, Monika Bowszyc-Dmochowska, Elżbieta Paszyńska, Dorota Jenerowicz

**Affiliations:** 1 Department of Oral Surgery, Periodontal Diseases and Oral Mucosal Diseases, Poznan University of Medical Sciences, 61-701 Poznan, Poland; ayakraiz@gmail.com; 2 Department of Dermatology, Poznan University of Medical Sciences, 61-701 Poznan, Poland; mbowdmo@ump.edu.pl (M.B.-D.); djenerowicz@ump.edu.pl (D.J.); 3 Department of Integrated Dentistry, Community Dentistry Section, Poznan University of Medical Sciences, 61-701 Poznan, Poland; paszynska@ump.edu.pl

**Keywords:** Miescher’s cheilitis, granulomatous cheilitis, intralesional corticosteroids, orofacial granulomatosis, Melkersson–Rosenthal syndrome, lip swelling

## Abstract

Miescher’s cheilitis (MC) is a rare, idiopathic inflammatory condition marked by recurrent or persistent swelling of the lips and adjacent orofacial areas. This case study aims to explore the clinical presentation of Miescher’s cheilitis and evaluate the effectiveness of intralesional corticosteroid therapy as a treatment approach. A 58-year-old male presented with severe, persistent swelling of both the upper and lower lips, which had been ongoing for six months. The initial treatment with chloroquine was discontinued due to adverse effects and no efficacy. Subsequent treatment involved intralesional injections of triamcinolone acetonide, administered at concentrations of 10 mg/mL and 40 mg/mL. After a total of ten injection sessions, the patient experienced a nearly 70% reduction in lip swelling, with the therapeutic effect lasting for 9 months. Intralesional corticosteroid therapy proved to be an effective treatment for Miescher’s cheilitis, offering significant symptom relief and improvement in lip swelling when other treatments were ineffective or unsuitable. This case highlights the need for individualized treatment plans and underscores the importance of ongoing research to refine management strategies for this challenging condition.

## 1. Introduction

Granulomatous cheilitis (GC), also known as Miescher’s cheilitis (MC), is a rare idiopathic inflammatory disorder characterized by recurrent or persistent, painless swelling of the lips and adjacent orofacial areas. The estimated prevalence of GC in general population is 0.08% [1]. It is often considered a monosymptomatic form of orofacial granulomatosis (OFG) or Melkersson–Rosenthal syndrome (MRS). However, the association between GC, OFG, and MRS is not always clear, and some clinicians treat them as separate conditions [2,3].

OFG was initially described as idiopathic, persistent, or recurrent labial swelling without identifiable systemic manifestations [4]. Besides the lips, OFG can affect intraoral areas including the oral mucosa, gums, tongue, pharynx, and larynx [5]. Swelling of the soft facial tissues is observed in 75–100% of patients diagnosed with OFG [6]. The swelling typically appears suddenly and lasts for a few days, but over time, it may become chronic. When OFG occurs alongside recurrent facial palsy, the condition is known as Melkersson–Rosenthal syndrome [7]. Peripheral facial nerve palsy affects 30–90% of patients [6], presenting as one-sided paralysis, which can be either partial or complete. A fissured tongue, the third symptom, is seen in 30–77% of patients [6]. The classic triad of MRS is present in 8–25% of cases [8]. GC is the most common presentation of MRS, observed in approximately 80% of cases [9]. The oligosymptomatic form, which includes lip swelling with fissured tongue, is the second most common manifestation of OFG [10]. The lack of a clear and consistent definition and classification of the disease complicates diagnosis and treatment [11]. There is no universally recommended treatment algorithm [12]. The diverse range of symptoms and their varying onset make GC of interest to various specialists, including dentists [13]. A dentist can play an integral role as part of a multidisciplinary team in the diagnosis and treatment of GC.

This study presents a case of a patient with Miescher’s cheilitis successfully treated with intralesional injections. Emphasis is placed on exploring potential treatment methods and the challenges associated with their application in clinical practice.

## 2. Case Presentation

A 58-year-old male patient was admitted to the Department of Dermatology at Poznan University of Medical Sciences with severe swelling of both the upper and lower lips. The first episode of lip swelling occurred 6 months before and lasted for 1 week, resolving spontaneously without treatment. A second episode began about 3 weeks after the first one and persisted for 6 months until hospital admission. The swelling was reported to be progressively enlarging. The patient reported no previous surgical, dental, or cosmetic procedures in the affected areas. He did not experience shortness of breath, throat obstruction, or urticaria. Family history was negative for Melkersson–Rosenthal syndrome and angioedema. The patient had no history of fever, gastrointestinal symptoms, fatigue, or weight loss. There were no known drug or food allergies. There was no prior treatment with antihistamines or steroids. The patient was wearing a partial upper removable denture but reported no related complaints. He did not report any taste disorders, impaired salivary secretion, or speech difficulties. The patient had a medical history of uncontrolled diabetes with diabetic retinopathy, hypertension, obesity, hyperlipidemia, and ischemic heart disease. Diabetes and hypertension were diagnosed ten years ago. Ischemic heart disease was diagnosed four years ago. He was taking rosuvastatin 40 mg/daily, bisoprolol 5 mg/daily, acetylsalicylic acid 75 mg/daily, fenofibrate 215 mg/daily, amlodipine, valsartan and hydrochlorothiazide 5 mg+160 mg+12.5 mg/daily, and insulin.

**Figure 1:**
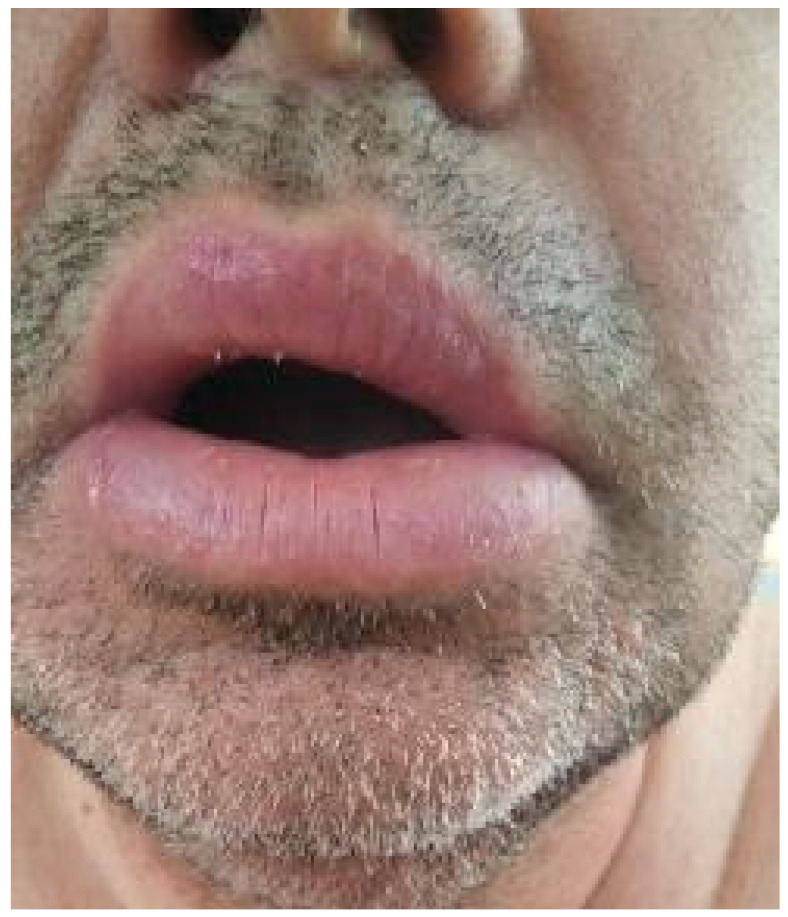
Swelling of the lips.

Upon examination, the patient exhibited severe swelling of the lower lip, less pronounced swelling of the upper lip, and swelling in the left buccal area. The lips were indurated, firm, non-tender, and diffusely swollen and presented with areas of scaling and dryness (Figure 1).

The tongue was normal on examination, and there was no regional lymphadenopathy. The facial nerve was also normal on examination.

Complete blood counts were within normal limits. The erythrocyte sedimentation rate (ESR) was 16 mm/h, and the C-reactive protein (CRP) level was 5.1 mg/L. Alanine aminotransferase (ALT) and aspartate aminotransferase (AST) levels were elevated at 57 U/L and 49 U/L, respectively. Sodium, potassium, and magnesium levels were normal. Laboratory analysis of urine revealed no abnormalities.

**Figure 2:**
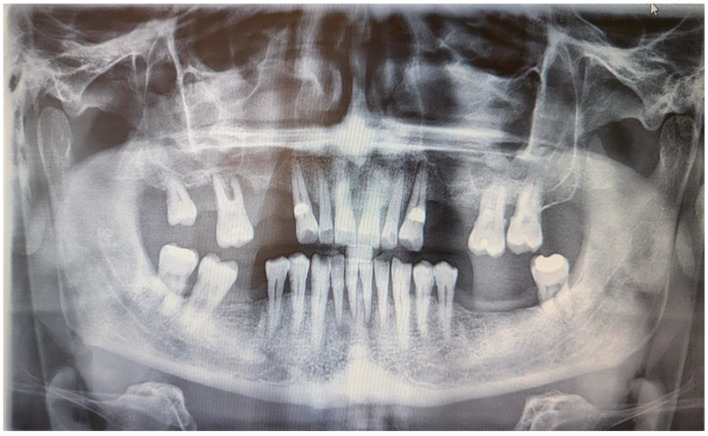
Digital OPG of the patient.

Skin prick tests for airborne and food allergies were negative, as were epidermal prick tests. Tuberculosis, sarcoidosis, and HBV and HCV infections were excluded. An abdominal ultrasound showed grade II fatty liver disease without liver enlargement. Chest X-ray was normal. A rheumatological consultation ruled out systemic connective tissue disease and other inflammatory musculoskeletal conditions, with an antinuclear antibody (ANA) titer of 1/80. A laryngological consultation found no signs of laryngological infection, and neurological examination revealed no peripheral facial nerve palsy. A dental examination conducted at the Department of Oral Surgery, Periodontal Diseases, and Oral Mucosal Diseases at Poznan University of Medical Sciences showed no signs of a fissured or plicated tongue. However, an intraoral examination revealed mild overgrowth of the gingival papillae and marginal gingiva. According to the Research Diagnostic Criteria for Temporomandibular Joint Disorders (RDC/TMD), there were no abnormalities in the temporomandibular joint or masticatory muscles, and no acoustic disturbances were detected in the temporomandibular joints. Additionally, there were no symptoms of stomatitis. Orthopantomographic examination showed significant bone loss around tooth 16 and less prominent bone loss in the lateral compartments of both maxilla and mandible. Moreover, the OPG showed discreet periapical radiolucency around mandibular incisors. There were no bone lesions. There were no defects of TMJ and maxillary sinus on both sides (Figure 2).

**Figure 3:**
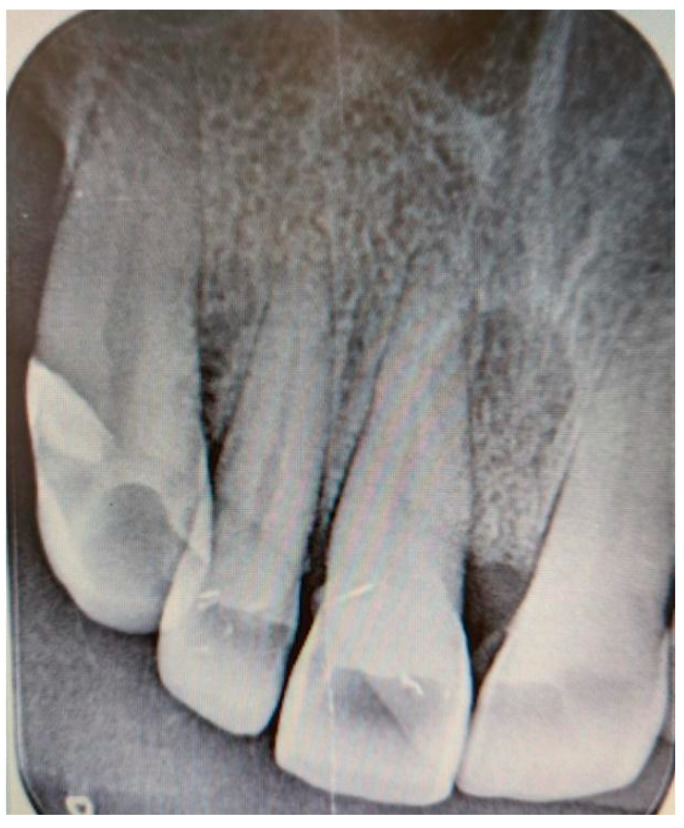
Digital periapical radiograph of the patient’s maxillary incisors.

**Figure 4:**
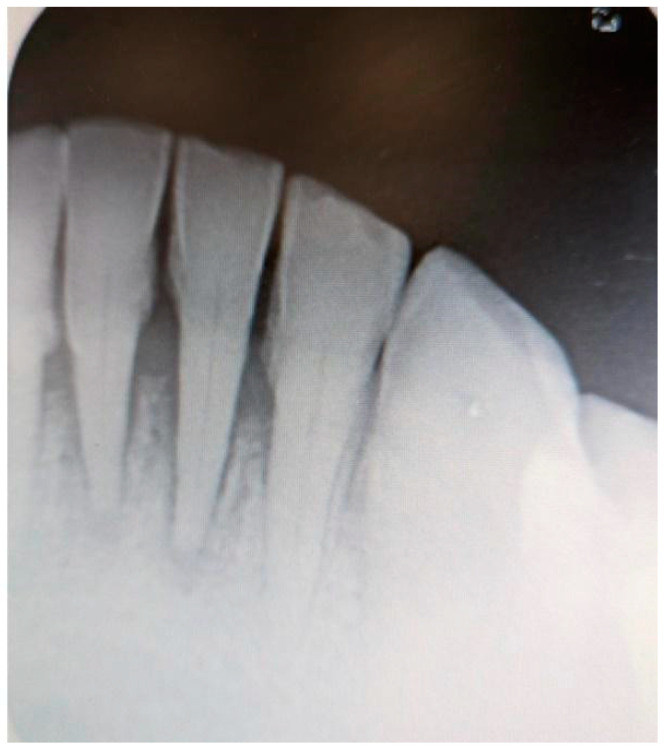
Digital periapical radiograph of the patient’s mandibular incisors.

Additionally, digital periapical radiographs of the patient’s maxillary and mandibular incisors were taken to exclude periapical lesions in these areas (Figure 3 and Figure 4).

Pulp vitality tests were conducted on teeth 11, 12, 32, 31, 41, and 42, all of which were found to be vital. No other dental disorders or potential odontogenic infections were identified during the examination.

**Figure 5:**
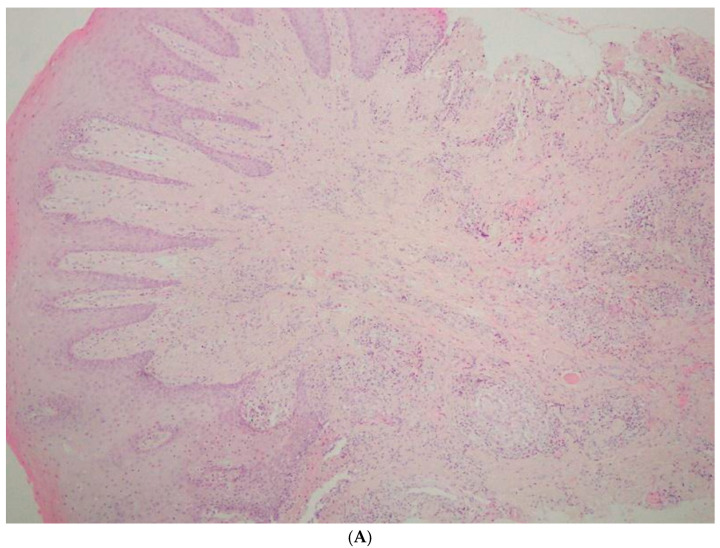

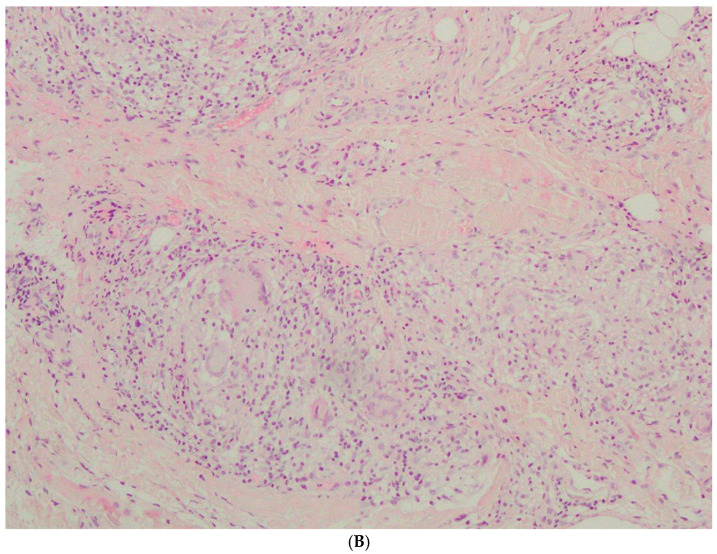
(**A**) Poorly marginated small granulomas in the middle and deep part of lamina propria of the mucosa of the lip (H + E staining, original objective magnification 4×). (**B**) Granuloma architecture with giant cells and epithelioid cells in the middle surrounded by lymphocytes (H + E staining, original objective magnification 40×).

Finally, an excisional biopsy of the lower lip was performed under local anesthesia, confirming the diagnosis of granulomatous cheilitis (Figure 5A,B). Indurated erythema of the face was ruled out.

The initial treatment was implemented at the Department of Dermatology. Due to the patient’s uncontrolled diabetes and related complications, systemic glucocorticoid therapy was not initiated. Instead, the patient was started on chloroquine at a dose of 200 mg once daily. An ophthalmologist’s consultation confirmed that there were no contraindications for chloroquine or hydroxychloroquine therapy. Additionally, tacrolimus 0.1% was applied topically, but without any effect. Local emollients were recommended to prevent lip cracking and dryness, along with sun protection. However, due to poor tolerance and a lack of significant improvement with chloroquine, the treatment was discontinued. The patient also reported peripheral nerve conduction issues during chloroquine therapy. Given the lack of response to pharmacological treatments, the patient was referred back to the Department of Oral Surgery, Periodontal Diseases, and Oral Mucosal Diseases to initiate intralesional treatment. Moreover, extraction of tooth 16 was recommended, but the patient declined the procedure. Scaling was performed, and the patient was provided with oral hygiene instructions.

**Figure 6:**
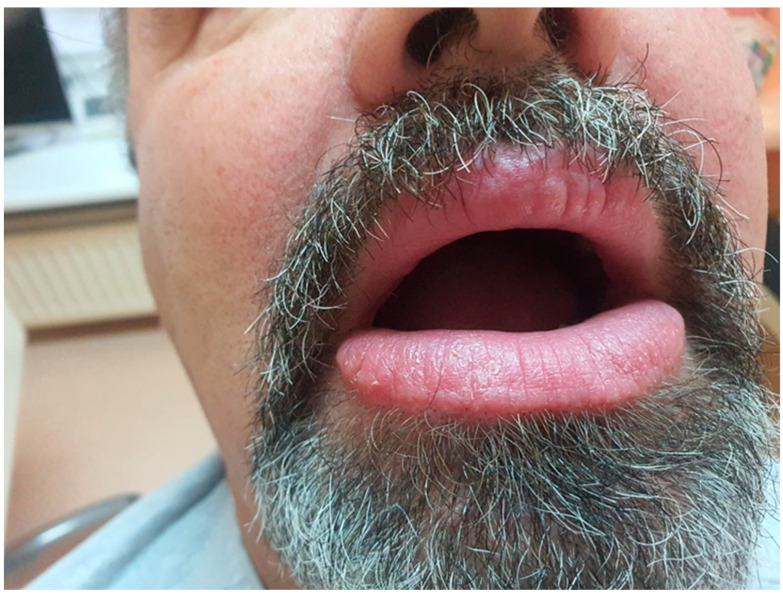
Effect of the therapy after 5 injections.

Subsequently, intralesional treatment of the lower lip was initiated. The patient received intralesional injections of triamcinolone acetonide at a concentration of 10 mg/mL, combined with lidocaine in a 1:5 ratio. This solution was symmetrically injected into both sides of the lower lip in equal volumes, with 4–6 injections per session. The same technique was used consistently for each session. A total of five injections were administered, with intervals of 2–3 weeks between sessions. Follow-up evaluations revealed an approximate 30% reduction in lower lip swelling (Figure 6).

**Figure 7:**
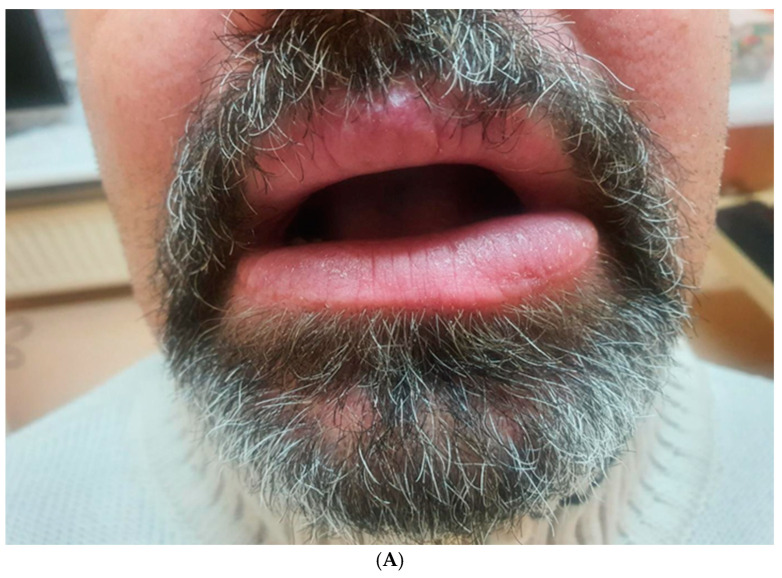

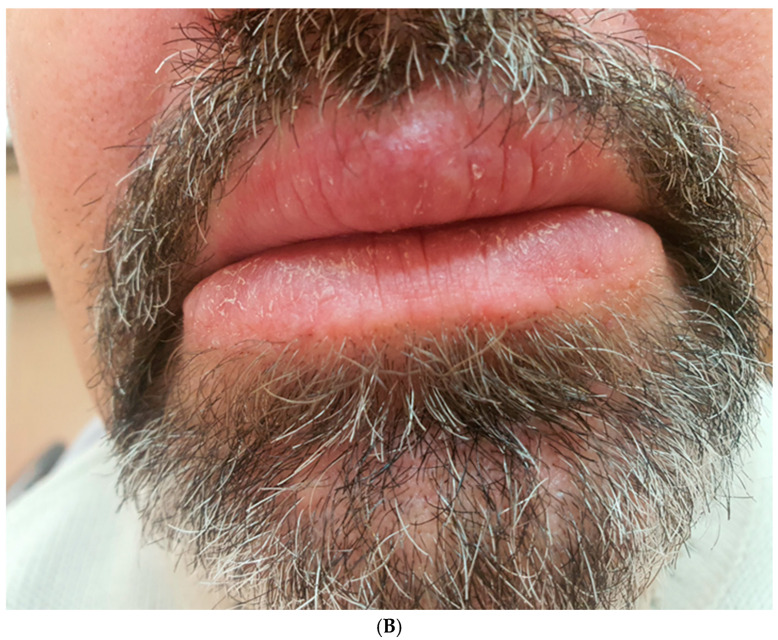
(**A**,**B**) Effect of the therapy after 8 injections with visible asymmetry between left and right size of the lower lip.

To prevent recurrence of swelling and achieve a better cosmetic outcome, we began administering intralesional injections of triamcinolone at a higher concentration (40 mg/mL). This approach resulted in a gradual reduction in lower lip size (Figure 7A,B).

**Figure 8:**
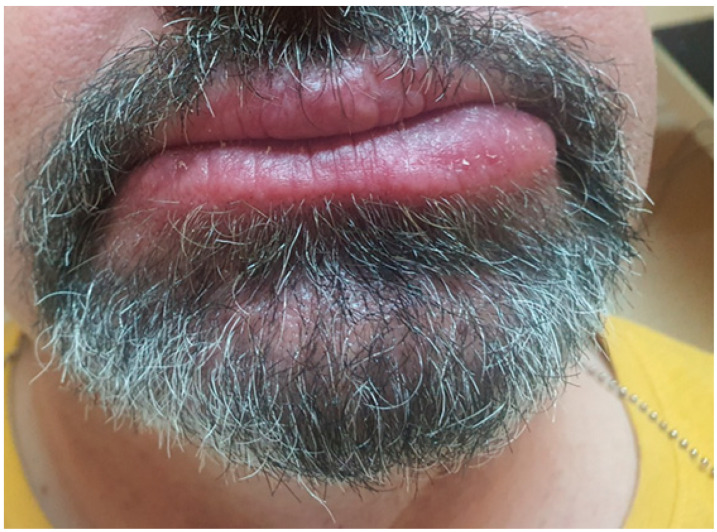
Effect of the therapy after 9 months.

After a total of 10 sessions—comprising 5 injections of triamcinolone acetonide at 10 mg/mL and 5 injections at 40 mg/mL—we achieved nearly a 70% reduction in the size of the lower lip. The initial effect of the therapy lasted for 9 months (Figure 8).

**Figure 9:**
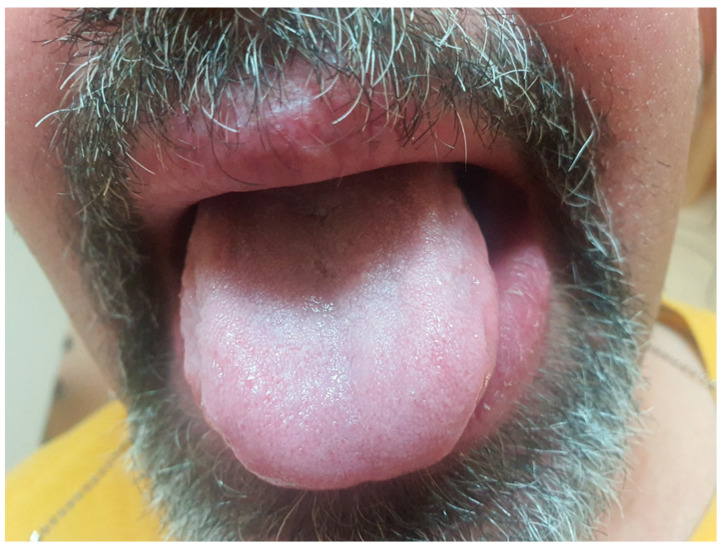
The patient presented no symptoms of a fissured tongue.

There were no signs of a fissured or plicated tongue for the whole disease duration, as presented in Figure 9.

**Figure 10:**
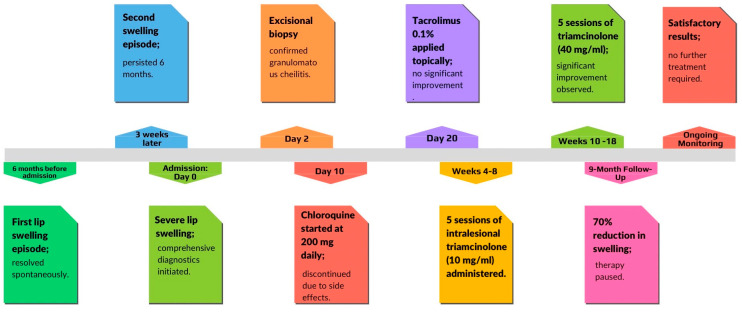
Chronological treatment timeline for MC patient case.

The patient is under continuous follow-up. Given the satisfactory cosmetic results and the significant reduction in the size of the lower lip, the decision was made not to continue with intralesional injections. The summarized data related to the patient’s treatment are presented in Figure 10.

## 3. Discussion

The diagnosis of MC is based on clinical symptoms and histopathological examination of lip tissue specimens, often requiring the collaboration of various medical specialties, including dentistry. As demonstrated in the presented case, dentists can play a vital role in treatment by identifying potential odontogenic infections in the oral cavity, assessing the oral mucosa, and implementing intralesional therapy. Additionally, dentists can help identify or rule out allergies to dental materials, such as amalgam fillings and hygiene products, as well as contact allergens that may be potential triggers for MC [14,15].

Due to unclear etiology, the treatment of MC is challenging and carries a high risk of ineffectiveness or recurrence. Additionally, many of the therapies implemented are based on single case reports and experiences of individual practitioners. There is currently no universally accepted algorithm for treating MC. Generally, pharmacological therapy is recommended as the initial treatment, preferred over surgical intervention [16]. Early conservative treatment is essential to prevent swelling from becoming permanent [17]. Delays in initiating therapy can reduce its effectiveness and increase the likelihood of persistent edema [18]. Surgical procedures are typically reserved for cases that do not respond to pharmacological treatment or for patients with frequent recurrences resulting in persistent or chronic edema [19]. Furthermore, surgical interventions are recommended for patients who do not show an active stage of the disease to avoid the stimulating granulomatous inflammatory process. All surgical procedures used in the treatment of MC aim at reducing the size of the lips.

Previous studies have explored various conservative treatment methods for MC, considering bacterial infections as one of the potential causes. Consequently, antibiotics are often administered during the first episode of lip swelling, provided that an allergic cause of the edema has been ruled out. However, these antibiotics typically do not yield the expected therapeutic effects and are rarely used as standalone treatments; they are generally combined with other medications during subsequent episodes of the disease [1]. Moreover, it is hard to assess how effective antibiotics used as a standalone therapy are in the initial episode of MC or to definitively exclude that swelling could disappear as a result of spontaneous remission. The standalone treatment with antibiotics includes amoxicillin–clavulanic acid and tetracyclines such as lymecycline and macrolides such as azithromycin and roxithromycin [5,20,21]. The best results were observed after standalone therapy with lymecycline and roxithromycin [20,21]. Currently, antibiotics are implemented as additional therapy in cases of lip swelling recurrence. They are usually combined with intralesional injections with triamcinolone, as presented in Table 1 [1,5]. Some antibiotics, including oral minocycline at a dose of 100 mg once daily and azithromycin pulse therapy (500 mg for three consecutive days, administered weekly), may be used together with intralesional steroid injections [1] as presented in Table 1. Intralesional steroids injections might be also combined with metronidazole [22]. Some clinicians also advocate for the use of tetracyclines, as their effectiveness in treating MC is attributed to their in vitro ability to inhibit granuloma formation by blocking protein kinase C. The combination of minocycline and steroids may help reduce the risk of edema recurrence [23]. Finally, tetracyclines are used postoperatively after cheiloplasty.

A recommended therapeutic approach involves infliximab combined with metronidazole or minocycline as well as anti-leprosy antibiotics such as clofazimine. Clofazimine is an interesting alternative to steroids. It is a relatively safe drug if used in short courses and in low doses. Clofazimine at a dose of 100 mg four times weekly for 3–11 months might trigger remission of moderate lip swelling. Doses of clofazimine range from 100 mg daily for 1 month to 100 mg three times a week for 3 months [24]. It is an effective and relatively low dose and can be given for a time period of up to 6 months with low toxicity and few side effects. Further treatment cycles are possible at any time after an interval of approximately 3 months. One of the most common side effects of clofazimine is red discoloration of the skin [24].

In addition to traditional antibiotics, other antibacterial agents are employed in the treatment of MC. Dapsone is a promising agent known for its antibacterial and anti-inflammatory properties; it suppresses neutrophil recruitment and the production of toxic secretory products. Dapsone enhances the efficacy of steroids, allowing for a reduction in their dosage, and is especially recommended for patients with contraindications to steroids. A suggested treatment regimen with dapsone involves a dosage of 100 mg/day for 2 weeks, followed by 50 mg/day for 25 weeks [25].

Another therapeutic option in MC is glucocorticoids. Some clinicians recommend long-term treatment with topical glucocorticoids including triamcinolone or clobetasol in orobase in less severe lip swelling [26]. In turn, topical triamcinolone or clobetasol in orobase may cause atrophy. In more severe lip swelling, systemic or intralesional glucocorticoids should be implemented. Systemic corticosteroids are frequently utilized due to their anti-inflammatory effects [27]. However, long-term use is not recommended, and they are contraindicated in compromised patients. Some clinicians affirm their effectiveness in cases of MC accompanied by severe swelling of the buccal mucosa, fissures, and gingival hyperplasia [28]. An effective alternative to systemic steroid use is intralesional steroid administration. Intralesional corticosteroids, such as triamcinolone acetonide, have been promising in localized treatment and management of MC [29]. They are most effective in the early stages of the disease, with efficacy diminishing over time. Triamcinolone injections are preferred due to their potent anti-inflammatory effects, being eight times more effective than prednisone and resulting in less sodium retention than with hydrocortisone therapy. A significant limitation of its use, however, is the potential for a considerable increase in the volume of the injected lip, complicating drug administration. Therefore, it is more beneficial to use smaller volumes of the drug at higher doses, a strategy confirmed in various studies, as higher doses help reduce the risk of recurrence [14].

The combination of intralesional steroids with local emollients and sun protection can enhance therapeutic outcomes and prevent recurrence [24,28,29].

Other pharmacological approaches include the use of disease-modifying antirheumatic drugs (DMARDs), which may be considered depending on the response to initial treatments [30]. Chloroquine and hydroxychloroquine have been investigated for their anti-inflammatory properties in similar conditions [31,32]. The case report presented in our study indicated that chloroquine and hydroxychloroquine may trigger some adverse effects especially in compromised patients and may be poorly tolerated by patients. On the other hand, the authors of this report have had a positive experience with these medications in a patient with ankylosing spondylitis. The 10-month infliximab initial therapy brought satisfactory improvement of the patient’s dermatological condition [6]. It was then decided to add chloroquine to the treatment. Chloroquine belongs to the group of antimalarials that show immunomodulatory, anti-inflammatory, antiproliferative, and photoprotective effects. With the accumulation of antimalarials in lysosomes, the pH increases. This inhibits the formation of antigenic peptides with class II molecules of the major histocompatibility complex, requisite to stimulate CD4+ T, and as a result, the immune response against autoantigenic peptides is suppressed.

Recent studies have underscored the efficacy of biologic agents, such as anti-TNF-alpha and anti-IL-17 medications, in treating inflammatory granulomatous conditions, suggesting these agents as potential alternative therapies for refractory cases [33,34]. These medications are used in both patients with full-blown presentations and those with monosymptomatic forms, such as MC. Infliximab and adalimumab are the primary biologic agents utilized in the treatment of MRS, demonstrating effectiveness even in the most persistent forms of edema. The recommended dosage for Infliximab ranges from 3 mg/kg to 5 mg/kg administered weekly, while Adalimumab is given at a dose of 40 mg per week [6]. A new group of biologic agents with suggested effectiveness in MC treatment are Janus-kinase (JAK) inhibitors such as Upadacitinib. Most patients treated with high-dose (30 mg/d) Upadacitinib presented clinical response within a median follow-up of 7.2 months. Upadacitinib was effective in MC combined with Crohn’s disease [35]. Moreover, some immunomodulators could be used in MC treatment. One of them is Thalidomide. It exerts specific inhibitory action on TNF-alpha. It decreases the responsiveness of polymorphonuclear cells to chemotactic factors. Due to its possible severe side effects, routine laboratory parameters including red and white blood cell counts and liver transaminase levels, neurologic status, and clinical findings should be regularly monitored during administration of thalidomide. In the therapy of MC, thalidomide is administered at a dose of 100 mg daily for 6 months and gradual reduction in the dose to 100 mg every other day for 2 months [33,36]. Some previous studies suggested the use of other immunomodulators and immunosuppressants such as methotrexate. The doses of methotrexate ranges from 5 to 10 mg orally, administered weekly [33].

Recent studies have shown good and long-term therapeutic results after localized therapy with photobiomodulation (PBM) using a diode laser with a 635 nm and 980 nm dual-wavelength (λ) approach, a 600-micron fiber, and a handpiece with a 1 cm-diameter lens at 300 mW. The overall treatments included three PBM sessions a week, administered for 4 weeks [3]. A reduction in lip size of about 35% was achieved.

Surgical treatment should only be performed in unresponsive and severely disfiguring cases. Moreover, any surgical intervention should be carried out in a quiescent phase of the disease. All surgical approaches including bermellectomy and Conway’s technique are based on the reduction in the lip size [2,4,37]. Conway’s technique is tailored to the most severe lip swelling and associated with both extraoral and intraoral flap harvesting [4,36]. Surgical treatment is especially recommended for monosymptomatic cases of the Melkersson–Rosenthal syndrome, limited to lip swelling. It gives a reliable and cosmetic solution for the morphological manifestation of the disease with positive both functional and esthetical outcome self-perceived by the patient. Although it allows a reliable and long-term effect, it does not prevent possible MC recurrence. To reduce the risk of swelling recurrence, some clinicians recommend additional intralesional injections with steroids immediately after surgical procedure or suggest the administration of antibiotics in the postoperative period [38].

The summarized data related to treatment of MC are presented in Table 1.

**Table 1:** Summarized data regarding treatment of MC.

Type of Treatment	Characteristics of the Treatment
**Antibiotics:** Short sole therapy Combined with intralesional injections with steroids Combined with intralesional injections with infliximab	Amoxicillin–clavulanic acid (625 mg twice daily for 1 week) [5] Tetracyclines: lymecycline [20] Macrolides: azithromycin at a dose of 1500 mg/week, roxithromycin at a dose of 150 mg/day [5,21] or 150 mg to 300 mg daily [33] Azithromycin pulse therapy (weekly azithromycin 500 mg for three consecutive days) for a month [1] Tetracyclines: doxycycline (200 mg once daily), minocycline (100 mg once daily) [5] Metronidazole: 750–1000 mg daily [5,22,33] Amoxicillin–clavulanic acid (625 mg twice daily for 1 week) [5] Metronidazole, Minocycline
**Anti-leprosy drugs**	Clofazimine dosages between 100 mg every other day to 100 mg per day [24] Dapsone 100 mg/day for 2 weeks, then 50 mg/day for 25 weeks [25]
**Topical glucocorticoids**	Long-term topical treatment with triamcinolone in orobase or clobetasol in orobase [26]
**Systemic glucocorticoids**	Prednisolone (5 mg twice daily for 10 days) [4] Methylprednisolone mg/kg bw in tapering doses over 3 to 8 weeks
**Intralesional glucocorticoids:** A sole therapy Combined with antibiotics or dapsone	Intralesional triamcinolone acetonide at 10 mg/mL [1] Intralesional triamcinolone acetonide at 40 mg/mL Intralesional triamcinolone at 40 mg/mL combined with oral minocycline 100 mg once daily [1] Intralesional betamethasone at 7 mg/mL: 1 mL per injection once a month for three months combined with a single daily dose of doxycycline 200 mg for 3 months [23] Intralesional triamcinolone and dapsone [5]
**Immunosuppressants/Immunomodulators**	Azathioprine Methotrexate 5–10 mg administered orally on a weekly basis [33] Thalidomide (100 mg/d for 6 months to 100 mg every other day for 2 months) [5] or 100 mg daily every other day [33,35] Tacrolimus
**Antimalarials/disease-modifying antirheumatic drugs (DMARDs)**	Chloroquine [2,30,31] Hydroxychloroquine [2,30,31]
**Antibodies monoclonal anti-TNF-α**	Infliximab (3 mg/kg–5 mg/kg per infusion) [6] Adalimumab (40 mg per week) [6]
**Photobiomodulation**	Photobiomodulation (PBM) using a diode laser with a 635 nm and 980 nm dual-wavelength (λ) approach [3]
**Surgical treatment**	Reducing cheiloplasty through partial bermellectomy of the lip [2] Conway’s technique of cheiloplasty [4,36] Cheiloplasty combined with intralesional injections with triamcinolone immediately after surgery [37] Cheiloplasty combined with administration of antibiotics in postoperative period: 500 mg tetracycline hydrochloride for 2 months, then 500 mg/d for 3 months and, finally, 250 mg daily for 6 months [37]

Differential diagnosis of MC is based on the duration and type of lip swelling. Reversible types of cheilitis such as simplex cheilitis and exfoliative cheilitis are characterized by less severe lip swelling and could result from local irritation, systemic immune suppression, nutritional deficiencies, or a reduction in lip moisture. Simplex cheilitis presents as cracked lips, fissures, or desquamation of the lips. Another manifestation of cheilitis is angular cheilitis. It is combined with fungal or bacterial infection. Angular cheilitis is one of the most common oral mucosal lesions in Sjögren’s syndrome and Sjögren’s-syndrome-related xerostomia. Moreover, it could be associated with immune and nutritional deficiencies including iron and riboflavin deficiencies. MC should be differentiated from contact cheilitis resulting from climatic, mechanical irritants, caustic agents, or allergens and develops as allergic contact cheilitis. A more persistent type of cheilitis is glandular cheilitis. It is a rare chronic inflammation of labial salivary glands, predominantly of the lower lip. Smoking, poor oral hygiene, chronic exposure to external influences such as sunlight, wind, tobacco, bacterial infection, and congenital predisposition are considered main etiologic factors of glandular cheilitis. Differential diagnosis of MC should include elephantiasis nostras, recurrent erysipelas and herpes simplex, macrocheilia, angioedema, Ascher’s syndrome, a foreign body reaction, Crohn’s disease, amyloidosis, organized hematoma, and other inflammatory diseases, especially specific inflammations including tuberculosis, Wegener’s granulomatosis, and sarcoidosis [8,39,40,41].

## 4. Conclusions

Miescher’s cheilitis presents diagnostic and therapeutic challenges due to its rare and variable clinical manifestations. This case report illustrates that intralesional corticosteroid injections, particularly with triamcinolone acetonide, are highly effective, especially when systemic treatments are contraindicated or poorly tolerated. The success of this approach emphasizes the importance of individualized treatment plans tailored to each patient’s specific needs.

Despite advancements in managing MC, a standardized treatment algorithm remains elusive due to the condition’s heterogeneous nature and its associations with orofacial granulomatosis and Melkersson–Rosenthal syndrome. Continued research is crucial to better understand the pathophysiology of these disorders and developing evidence-based treatment guidelines.

The case underscores the need for a multidisciplinary approach involving dermatologists, oral surgeons, and other specialists to comprehensively address the patient’s condition, ultimately improving outcomes and quality of life. Future studies should aim to optimize treatment protocols and explore new therapeutic options, including biologic agents and advanced imaging techniques.

In summary, while intralesional corticosteroids show promise for treating MC, refining diagnostic criteria and treatment strategies will be essential in managing this complex and rare disorder.

## Data Availability

The data presented in this study are available on request from the corresponding author due to ethical reason.
